# The dysbiosis signature of *Fusobacterium nucleatum* in colorectal cancer-cause or consequences? A systematic review

**DOI:** 10.1186/s12935-021-01886-z

**Published:** 2021-04-06

**Authors:** Maryam Ranjbar, Rasoul Salehi, Shaghayegh Haghjooy Javanmard, Laleh Rafiee, Habibollah Faraji, Sima jafarpor, Gordon A. Ferns, Majid Ghayour-Mobarhan, Mostafa Manian, Reza Nedaeinia

**Affiliations:** 1 Applied Physiology Research Center, Cardiovascular Research Institute, Isfahan University of Medical Sciences, Isfahan, Iran; 2 Department of Genetics and Molecular Biology, School of Medicine, Isfahan University of Medical Sciences, Isfahan, Iran; 3 Pediatric Inherited Diseases Research Center, Research Institute for Primordial Prevention of Non-Communicable Disease, Isfahan University of Medical Sciences, Isfahan, Iran; 4 Molecular Medicine Research Center, Hormozgan Health Institute, Hormozgan University of Medical Sciences, Bandar Abbas, Iran; 5 Division of Medical Education, Brighton and Sussex Medical School, Falmer, Brighton, Sussex, BN1 9PH UK; 6 Metabolic Syndrome Research Center, Mashhad University of Medical Sciences, Mashhad, Iran; 7 Child Growth and Development Research Center, Research Institute for Primordial Prevention of Non-Communicable Disease, Isfahan University of Medical Sciences, Isfahan, Iran

**Keywords:** Colorectal cancer, Dysbiosis signature, *F. nucleatum*, Gut microbiota

## Abstract

Colorectal cancer (CRC) is the third most common cause of cancer globally and the fourth attributable cause of mortality and morbidity due to cancer. An emerging factor contributing to CRC is the gut microbiota and the cellular changes associated with it. Further insights on this may help in the prevention, diagnosis and new therapeutic approaches to colorectal cancer. In most cases of CRC, genetic factors appear to contribute less to its aetiology than environmental and epigenetic factors; therefore, it may be important to investigate these environmental factors, their effects, and the mechanisms that may contribute to this cancer. The gut microbiota has recently been highlighted as a potential risk factor that may affect the structural components of the tumor microenvironment, as well as free radical and enzymatic metabolites directly, or indirectly. Many studies have reported changes in the gut microbiota of patients with colorectal cancer. What is controversial is whether the cancer is the cause or consequence of the change in the microbiota. There is strong evidence supporting both possibilities. The presence of *Fusobacterium nucleatum* in human colorectal specimens has been demonstrated by RNA-sequencing. *F. nucleatum* has been shown to express high levels of virulence factors such as *FadA*, *Fap2* and *MORN2* proteins. Our review of the published data suggest that *F. nucleatum* may be a prognostic biomarker of CRC risk, and hence raises the potential of antibiotic treatment of *F. nucleatum* for the prevention of CRC.

## Introduction

CRC is one of the most common malignancies of men and women in most countries [1, 2]. The third most common cause of cancer and the fourth leading cause of cancer-related deaths [3]. More than 1.2 million new cases of CRC are reported annually throughout the world [4]. Identification of the microbial mechanisms involved in the etiology of CRC and the recognition of the associated cellular changes as one of the factors in the development of cancer may contribute to cancer prevention, its early diagnosis and potentially new therapies for CRC. The trend is for a projected increase in CRC by 60% to more than 2.2 million new cases, and 1.1 million cancer deaths by the year 2030 [3]. This increase in prevalence has caused considerable debate about the most appropriate prevention approaches. These predictions represent a major problem in developing and developed countries in the public health sector [5]. There has been a global increase in the standardised rate of the age of CRC from 1990 to 2017 with considerable heterogeneity at regional and national level. There has been a decline in the age-standardized death and disability-adjusted life-years rates (DALY) [6]. According to estimates by the DALY criteria, CRC is the world's 36th leading cause of death from disease in 2017 and the fourth most common cause of cancer. The gut microbiota may be one environmental risk factor predisposing to CRC [7]. Bacteria are found throughout the human body, but especially in the digestive tract [8]. The gut at birth is sterile, but some organisms enter it soon after birth. In breastfeed infants, the gut contains a large number of *Lactic acid bacteria* and *Lactobacilli, streptococci*, for example *bifidobacterium*. The gut *microflora* changes with changes in dietary habits and the selection of adult dietary patterns [9]. Gut bacteria are important for the synthesis of vitamin K, and for the conversion of bile pigments and bile acids to secondary bile acids [10]. In addition, these bacteria are involved in the uptake of food and metabolic products and have antagonistic effects with microbial pathogens. The microbial flora of the gut produces ammonium and other metabolic products absorbed from the intestinal mucosa and can participate in the occurrence of hepatic coma. Anaerobic colonic bacteria, such as *Bacteroides fragilis*, *Clostridium* and *Peptostreptococcus* play a role in the progress of intra-abdominal abscesses [11]. Therefore, intestinal microbes appear to play a crucial role in digestive function and health [12, 13]. It has been proposed that commensal bacteria in the colon play a vital role in the development of CRC [14]. Various studies have shown that chronic infections can be important factors in the development of cancer. Gastric, liver, and cervical cancers are caused by *Helicobacter*, *Hepatitis B* and *C* and *human Papillomavirus*, respectively [15, 16]. These pathogens activate tumor signaling pathways like *NF-kB*, *STAT3* [15–17]. There is good evidence for a relationship between gut microbiota and CRC [18]. This is proposed to be due to the expression of proteins that have antiapoptotic, growth factor or cytokine that enhance cancer cell growth, metastasis or resistance to therapy [17]. However, *F. nucleatum* has been shown to also express high levels of virulence factors such as *FadA*, *Fap2* and *MORN2* proteins [19]. Studies have demonstrated that the dominant microbiome is very similar in primary and metastatic tumors [20]. It is assumed that *Fusobacterium* moves to distant sites with primary tumor cells as a part of metastatic tissue colonization. This indicates that the tumor microbiomes are the essential components of the cancerous microenvironment [20, 21].

## Objectives

In this paper, we aimed to examine the potential role of the gut microbiome, especially *F. nucleatum*, in inhibiting the immune system in CRC and the stimulatory effects of its surface proteins on the establishment or dissemination of CRC and stimulation of its tumorigenic signals. Also, the role of *F. nucleatum* and its virulence factors in the development of CRC in particular are systematic reviewed. The cellular signals associated with the creation of tumors activated by bacteria will also be explained.

## Search strategy

The protocol was performed in accordance with the preferred reporting items for Systematic reviews and Meta-Analyses (PRISMA) guidelines [22], outlined in Table 1. The following databases were searched: MEDLINE, Embase via PubMed, Scopus, Web of Science database and Google Scholar. A manual search was used to find reference lists of related articles and reviews. In order to locate reference lists of relevant publications and reviews, a manual search was used. The above manual search was made in order to find articles that were not identified by internet searches. The authors were consulted to collect further information in situations where it was needed. Language constraints have been imposed for the search or collection of English publications written in December 2020. The following key- words were used in this search: [(Colorectal[Title/Abstract] OR Intestinal[Title/Abstract]) AND (Neoplasm*[Title/Abstract] OR Carcinoma*[Title/Abstract] OR Cancer*[Title/Abstract] OR Tumor*[Title/Abstract] OR Malignanc*[Title/Abstract])] OR [Adenoma*[Title/Abstract] AND (Colon [Title/Abstract] OR Intestin* [Title/Abstract] OR colonic [Title/Abstract] OR Polypos* [Title/Abstract])] AND [(fecal[Title/Abstract] OR faecal[Title/Abstract] OR feces[Title/Abstract]) AND (Fusobacteri*[Title/Abstract] OR *F. nucleatum*[Title/Abstract])] AND [Microbio*[Title/Abstract] OR Microbial[Title/Abstract] OR Diet[Title/Abstract] OR Dysbios*[Title/Abstract] OR Dysbacterios*[Title/Abstract] OR MicroRNA[Title/Abstract] OR miRNAs[Title/Abstract] OR “Micro RNA” [Title/Abstract] OR miRNA[Title/Abstract]] OR [Marker*[Title/Abstract] AND (Tumor[Title/Abstract] OR Carcinogen*[Title/Abstract] OR Neoplasm*[Title/Abstract] OR Cancer[Title/Abstract])) OR (Biomarker*[Title/Abstract] AND (Tumor[Title/Abstract] OR Carcinogen*[Title/Abstract] OR Neoplasm*[Title/Abstract] OR Cancer[Title/Abstract])) OR immunomodulator*[Title/Abstract]].

**Table 1:** Characteristics of included studies that examine *F. nucleatum* in colorectal cancer patient

Authors	Year	Country	Details of study	Sample type (n)	Detection method	Main findings
Haruki et al. [23]	2020	Boston, USA	Analysis of Fn status in tumor tissue and evaluation autophagic activity of tumor cells by analysis SQSTM1, BECN1, and MAP1LC3 expression	Tissue (724)	qPCR	Fn was detected in 14% colorectal cancer cases High and intermediate expression of BECN1 gene in colorectal cancer tissue were inversely associated with the amount of Fn that suggested possible role of autophagy (Ptrend < 0.001) and the expression of SQSTM1 and MAP1LC3 in tumors were not significantly associated with the level of Fn (Ptrend > 0.06) No significant association was observed between the expression of BECN1, MAP1LC3, and SQSTM1 and patient survival (Ptrend > 0.10)
Okita et al. [24]	2020	USA	Assessment whether *F. nucleatum* status can be a carcinogenic factor and determine molecular characteristics of CRC	Tissue (304) CRC Japan (174)	qPCR	There are a significant association between microsatellite instability-high (MSI-H) and L/E and the amount of *F. nucleatum* (high and moderate) *F. nucleatum* infection induce DNA damage in colon tissues
Chen et al. [25]	2020	China	Investigation the relationship between *F. nucleatum* status and metastasis in CRC patient	Fecal (49) Tissue (83)	qPCR	There was a significant relationship between Fn infection and CRC metastasis so CRC patients with lymph nodes metastasis have high level of *F. nucleatum* infection *F. nucleatum* infection can increase KRT7-AS/KRT7 expression which induced cell migration in vivo and in vitro
Chen et al. [26]	2020	China	Assessment *F. nucleatum* status in CRC patients and investigation its role in tumor metastasis	Tissue (62)	FISH	*F. nucleatum* was significantly high in metastatic CRC compare to non-metastatic CRC (*P* = 0.01) *F. nucleatum* level was higher in metastatic lymph nodes than controls (*P* = 0.003)
Abed et al. [27]	2020	USA	Evaluation of *F. nucleatum* in CRC and assessment whether CRC-fusobacteria originate from the oral microbial	Saliva (7)	qPCR Immunofluorescence Hemagglutination Assay	Oral microbial are the source of CRC-fusobacteria Hematogenous fusobacteria were more successful in CRC colonization than gavaged ones in the MC38 and CT26 mouse orthotropic CRC models
Chen et al. [28]	2019	China	Investigation the relationship between F nucleatum and microsatellite instability status, clinicopathological features and its prognostic effect in CRC patient (stages II–III)	FFPE (91)	qPCR	No significant relationship was observed between *F. nucleatum* status and clinic pathological features (P > 0.05) F nucleatum species (OR: 2.094, 95% CI [1.178–8.122], P = 0.032) and MSI status (OR 2.243, 95% CI 1.136–5.865, P = 0.039) were independent prognostic factors in CRC patient
Feng et al. [13]	2019	China	Analysis of genes and miRNAs involved in the progression of *F. nucleatum*-induced CRC	Tissues (15)	miRNA microarray Human Transcriptome Array	miR-4717 and miR-4474 were significantly up-regulated in the tumor tissue compared to the normal in response to *F*.* nucleatum* infection Bioinformatic analysis revealed that CREB-binding protein (CREBBP) is the primary aberrantly expressed gene in *F. nucleatum*-induced CRC Real-time RT-PCR analysis showed that miR-4474/4717 was upregulated while CREBBP was downregulated in CRC patients with *F. nucleatum* infection CREBBP was introduced as a novel target of miR-4474/4717
Butt et al. [29]	2019	Europe	Assessment whether antibody responses to *F. nucleatum* are correlated with CRC risk in prediagnostic serum samples of patient	EPIC cohort: serum (485)	Multiplex serology method	No significant association was observed between antibody against *F. nucleatum* and colorectal cancer risk (OR, 0.81; 95% CI 0.62–1.06)
Guven et al. [30]	2019	Turkey	Examination the quantities of three CRC related bacteria such as *F. nucleatum*, etc. in CRC patients	Saliva (71)	qPCR	*F. nucleatum* amount was higher in Saliva samples of CRC patient compared to controls (P = 0.001) No significant results was observed in ROC curve analyses for *F. nucleatum*
Tunsjø et al. [31]	2019	Norway	Investigation the levels of *Fusobacterium* *nucleatum* in order to evaluate microbiome-based biomarkers for non-invasive detection of CRC	Stool and mucosa (72)	qPCR	Levels of *F. nucleatum* in stool samples were significantly higher in the cancer group compared with the the polyp group(P = 0.0028) and control group (P = 0.0073)
Kunzmann et al. [32]	2019	Czech Republic	Evaluation of *F. nucleatum* as a prognostic biomarker and assessing its association with post-diagnosis survival	Tissues (190)	qRT-PCR	*F. nucleatum* level was significantly high in the tumor tissue compared to the normal mucosa (P = 0.002) High levels of Fn was associated with poorer overall survival (HR 1.68, 95% CI 1.02–2.77, P = 0.04)
Zhang et al. [33]	2019	China	Assessment whether high expression of BIRC3 induced by *F. nucleatum* can be responsible for chemoresistance to 5-Fu in CRC patient	FFPE (94)	qPCR	*F. nucleatum* infection can upregulate BIRC3 expression by the TLR4/NF-κB pathway in CRC cells and decrease the chemosensitivity of cancer cells to 5-Fu in vitro and in vivo There was a significant correlation between high level of *F. nucleatum* and chemoresistance in high stage CRC patients treated by 5-Fu-based adjuvant chemotherapy
Lee et al. [34]	2018	Korea	Investigation the association between *F. nucleatum* status, patient prognosis and pathway mutation in CRC patient (stages II–III)	FFPE and Tissue (246)	qPCR	*F. nucleatum* amount was higher in CRC subjects compared to controls (P < 0.001) High levels of Fn was associated with poorer overall survival in metastatic CRC (P = 0.042) Mutation rate of AMER1 (P = 0.030), ATM (P = 0.008), and TGF-b pathway (P = 0.020) were associated with high amount of *F. nucleatum*
Yamaoka et al. [35]	2018	Japan	Measuring absolute copy numbers of *F. nucleatum*	Tissue (100)	droplet digital PCR	*F. nucleatum* was detected in 75.0% CRC tissues and significantly higher in CRC tissue than normal (P = 0.0031) Fn copy number (median) was 1.6 copies/ng DNA in CRC and 0.4 copies/ng DNA in normal group (P = 0.0046)
Hamada et al. [36]	2018	USA	Assessment the association of *F. nucleatum* in colorectal cancer tissue with immune response might differ by tumor MSI status	NHS and HPFS Cohorts: Tissue (1041)	qPCR	Negative association was observed between *F. nucleatum* level and tumor-infiltrating lymphocytes (TIL) in MSI-high tumors (OR: 0.45; 95% CI [0.22–0.92]) but positive association was observed between the presence of *F. nucleatum* and TIL in non-MSI-high tumors(OR: 1.91; 95% CI [1.12–3.25])
Chen et al. [37]	2018	China	Evaluation the association between the presence of *F. nucleatum* with CD4+ T-cell density and thymocyte selection-associated high-mobility group box (TOX) protein expression	Tissue (138)	IHC, FISH, Immuno-fluorescence	CD4+ T-cell density and TOX expression were higher in *F. nucleatum*-negative tissues compared *F. nucleatum*-positive tissues (P = 0.002, P < 0.001, respectively) Negative correlation was observed between *F. nucleatum* level and TOX expression (P < 0.001) and CD4+ T-cell density (P < 0.001) *F.* *nucleatum* may inhibit antitumor immune response by reduction in TOX expression and CD4+ T-cell density in the colorectal cancer
Liu et al. [38]	2018	USA	Examination inflammatory diet intakes in relation to incidence of colorectal cancer subtypes in response to *F*. *nucleatum* infection in tumor tissue	NHS and HPFS Cohorts: (951)	qPCR	Increased risk of *F. nucleatum*-positive colorectal tumors was associated with higher dietary inflammatory pattern (EDIP) score (Ptrend = 0.03) There was a significant associated between proximal *F. nucleatum*-positive colorectal tumors and High EDIP scores (Pheterogeneity = 0.003)
Guo et al. [39]	2018	China	Measuring the relative the quantities of *F. nucleatum* and several probiotics in of CRC Evaluation the diagnostic performance of these microbial ratios and investigation the bactericidal activity of *F. nucleatum* against probiotics	Stool Cohort I. CRC (215), BCD (178), NGC (100), 156 HCs Cohort II. CRC (152), 102 HCs	qPCR 16S rDNA sequencing	The sensitivity of 84.6% and specificity of 92.3% for in detecting CRC was calculated in the microbial ratio of *F. nucleatum* to *Bifidobacterium* *F. nucleatum* negatively correlated with *Fusobacterium nucleatum* in CRC patient *F. nucleatum* may have role in dysbiosis via the secreted antagonistic against *Bifidobacterium* and *Faecalibacterium prausnitzii*
Proença et al. [40]	2018	Brazil	Examination the effect of *F. nucleatum* on the microenvironment of colonic neoplasms and the expression of inflammatory mediators and miRNAs	Tissue sample CRC (43) CRA (27)	qPCR	*F. nucleatum* was detected in 51.8% CRA and in 72.1% CRC tissues *F. nucleatum* level was correlated with the expression of miR-22 (r = 0.38, P = 0.0331), IL8 (r = 0.54, P = 0.0013), IL1B (r = 0.46, P = 0.0066), IL6 (r = 0.47, P = 0.0059), and IL8 (r = 0.54, P = 0.0013) Positive correlations were observed between *F. nucleatum* level and miR-22 (r = 0.38, P = 0.0331), cytokines; IL8 (r = 0.54, P = 0.0013), IL1B (r = 0.46, P = 0.0066), IL6 (r = 0.47, P = 0.0059), and IL8 (r = 0.54, P = 0.0013) in the CRC group Negative correlation were observed between *F. nucleatum* level and TLR4 (r = − 0.62, P = 0.0235) in the CRA group The abundance of *F. nucleatum* was associated with KRAS mutation (P = 0.0432) in CRC samples
Chen et al. [41]	2017	China	Assessment the association between β-catenin nuclear accumulation and *F. nucleatum* infection in CRC patient and examination whether *F. nucleatum* infection can activate β-catenin signaling via the TLR4/P-PAK1/P-β-catenin S675 cascade in CRC patient	Tissue 98	FISH	No significant association was observed between *F. nucleatum* status and clinicopathologic features in CRC tissue (P > 0.05) *F. nucleatum* infection was higher in proximal CRCs than in distal CRCs (P = 0.045) The frequency of TLR4, PAK1 and nuclear β-catenin proteins were higher in Fn-positive than Fn-negative CRCs (P < 0.05) *F. nucleatum* significantly can increase TLR4/P-PAK1/P-β-catenin S675/C-myc/CyclinD1 proteins expression suggesting that *F. nucleatum* infection can active β-catenin in TLR4/PAK1 cascade and help to the carcinogenesis of CRCs
Yan et al. [42]	2017	China	Analysis the levels of Fn and its prognostic significance in human CRC (stage III/IV) and normal tissues	Tissues (280)	qPCR	Fn level is significantly higher in CRC tissues than in adjacent normal tissues (P < 0.001) High level of Fn was significantly correlated with lymph node metastasis status (P = 0.008), tumor invasion (P = 0.015), and distant metastasis (P = 0.020). Fn level was significantly correlated with the expression of E-cadherin (r = − 0.301, P < 0.001), N-cadherin N-cadherin (r = 0.377, P < 0.001), and Nanog (r = 0.362, P < 0.001) Patients with low level of Fn had a significantly better cancer-specific survival (CSS) and disease-free survival (DFS) than those with high Fn level (CSS, P < 0.001; DFS, P < 0.001)
Suehiro et al. [43]	2017	Japan	Developing a method for *F. nucleatum* detection in stool sample of CRC patient and investigation the association between *F. nucleatum* status in stool with the progression of colorectal cancer	Feces: CIS (19) CRC (158)	ddPCR	*F. nucleatum* level was higher in stool sample of CRC patient (P < 0.0001) and advanced adenoma/CIS group (P = 0.0060) than controls Droplet digital PCR has high sensitivity for detection of *F. nucleatum* in the stool sample of CRC patient
Ye et al. [44]	2017	Texas	Identification the specific *Fusobacterium* spp. and ssp. in clinical CRC specimens and assessment the behavior of colorectal cancer cells and monocytes in response to *F. nucleatum* infection in coculture systems	Tissue (25)	qPCR Cytokine panel assay, ELISA	*F. nucleatum* ssp. Animalis induced CCL20 expression in monocytes and colorectal cancer cells in In in vitro co-culture experiment *F. nucleatum* ssp. Animalis infection can induce inflammatory response and promote colorectal cancer
Yu et al. [45]	2017	China	Investigation the contribution of gut microbiota to chemoresistance in CRC patients	Cohort 1: Tissue (31) Cohort 2: FFPE (92) Cohort 3: FFPE (173)	qPCR	*F. nucleatum* amount was high in CRC patients with recurrence post chemotherapy and may promote chemoresistance by the Autophagy Pathway *F. nucleatum* -induced chemoresistance is regulated by MiR-18a* and miR-4802
Mehta et al. [46]	2017	USA	Assessment the associations of prudent and Western diets with colorectal cancer risk in response to *F*.* nucleatum* infection in tumor tissue	NHS and HPFS Cohorts: (137,217)	qPCR	The association between prudent diet and colorectal cancer risk significantly differed in *F*. *nucleatum* infection (Pheterogeneity = 0.01) Significant inverse correlation was observed between Prudent diet score and *F. nucleatum*-positive cancer risk (Ptrend = 0.003), but not with *F. nucleatum*-negative cancer risk (Ptrend = 0.47)
Amitay et al. [47]	2017	Germany	Examination the presence and relative abundance of *F. nucleatum* in fecal samples	Stool (500)	16S rRNA gene analysis	*F. nucleatum* level in feces was associated with the colorectal cancer (P < 0.0001)
Mima et al. [48]	2016	USA	Measuring the amount of *F. nucleatum* DNA in colorectal tumor tissue and analysis the relationship of a bowel subsite variable with *F. nucleatum* level	FFPE (1102)	qPCR	*F. nucleatum* DNA was detected in 13% of colorectal carcinoma tissue *F. nucleatum* status gradually increases from rectum(2.5%) to cecum(11%) in CRC with a significant trend along all subsites (P < 0.0001)
Nosho et al. [49]	2016	Japan	Analysis of Fn status in DNA samples from formalin-fixed paraffin embedded (FFPE) tissues in CRC patient (stages I–IV)	Tissues (511)	qPCR	Fn positivity in the Japanese patient was 8.6% which was lower than that in United States cohort studies (13%) Similar to the United States studies, Fn positivity in Japanese colorectal cancers was significantly associated with microsatellite instability (MSI)-high status. Regarding the immune response in colorectal cancer, high levels of infiltrating T-cell subsets (i.e., CD3+, CD8+, CD45RO+, and FOXP3+ cells) have been associated with better patient prognosis
Li et al. [50]	2016	China	Investigation the Fn abundance in tissues and its association with CRC	Tissues (101)	q-PCR FISH	Fn was over-represented in 87.1% of CRC tissues and Fn level is significantly higher in CRC tissues than in adjacent normal tissues (P < 0.001) *F. nucleatum* level was significantly higher in the lymph node metastases group than in the non-metastases group (P < 0.005)
Mima et al. [51]	2016	USA	Analysis of the association between *F. nucleatum* level and worse clinical outcome	FFPE (1069)	qPCR	*F. nucleatum* was detected in 13% CRC tissues *F. nucleatum* level is associated with shorter survival in CRC patient (Ptrend = 0.020) The level of *F. nucleatum* was associated with MSI-high (multivariable OR: 5.22; 95% CI 2.86 to 9.55)
Wang et al. [52]	2016	China	Measuring anti-Fn antibodies levels in CRC patients and evaluation of diagnostic value of serum anti-Fn antibodies in CRC patients	Stool (10) Serum (258)	PCR indirect whole-cell ELISA	Fn-infection can induce high level of anti-Fn antibodies in the serum of CRC patients Anti-Fn-IgA and -IgG were significantly higher in CRC patient than benign colon and control group (P < 0.001) Combination of anti-Fn-IgA with carcino-embryonic antigen (CEA) had diagnostic value CRC patient (Sen: 53.10%, Spe: 96.41%; AUC = 0.848)
Fukugaiti et al. [53]	2015	Brazil	Evaluation the presence of oral and intestinal microorganisms in the fecal microbiota of CRC patients and controls	Stool (17)	qRT-PCR	They were detected significantly more *F. nucleatum* in the Cancer Group than in the healthy Group (P = 0.01)
Mima et al. [54]	2015	USA	Assessment the hypothesis that *F. nucleatum* status in colorectal carcinoma is associated with lower amount of T-cells in tumor	NHS and HPFS Cohorts: FFPE (598)	qPCR Tissue microarray IHC	*F. nucleatum* was detected in 13% of colorectal carcinoma tissue Negative association was observed between *F. nucleatum* status and CD3+ T-cell density in colorectal carcinoma tissue OR, 0.47; 95% CI [0.26 to 0.87]; Ptrend = 0.006) No significant association was observed between *F. nucleatum* and density of CD8+, CD45RO+ , or FOXP3+ T-cells (Ptrend > 0.013)
Ito et al [55]	2015	Japan	Investigation *F. nucleatum* status in premalignant colorectal lesions and its association with CIMP, MSI and microRNA-31 status	FFPE (511)	qPCR	*F. nucleatum* was detected in CIMP-high premalignant lesions than in CIMP-low/zero lesions (P = 0.0023) *F. nucleatum* positivity was higher in CRCs (56%) than in premalignant lesions of any histological type (P < 0.0001)
Tahara et al*.* [56]	2014	Japan	Analysis of *F. nucleatum* (Fn) status and molecular features of tissue samples of colorectal cancer patient, colonic mucosae and control groups	Tissues (149)	q-PCR	Fn was detected in CRC tissues (74%) and the amount of Fusobacterial in normal tissue was 250-fold lower (mean) compared to CRC tissues Fn species in CRC group were associated with microsatellite instability (P = 0.018), CpG island methylator phenotype positivity (P = 0.001) and some genes: *TP53* wild type (*P* = 0.015), *hMLH1* methylation (P = 0.0028) *CHD7/8* mutation positivity (*P* = 0.002)
Flanagan et al. [57]	2014	Germany Czech Republic (CZ)	Evaluation of the potential of *F. nucleatum* as a biomarker for CRC by measuring survival outcomes and assessing its association with the adenoma to cancer progression	Tissue Czech cohort (49) German cohort (45) Irish cohort (28) adenoma (52) Stool CRC (7) adenoma (24)	qPCR	*F. nucleatum* amount was higher in cancerous than matched normal tissue (P < 0.0001) No significant association was observed in the *F. nucleatum* level between disease versus normal tissue (P = 0.06) in colorectal adenoma (CRA) Low Fn levels was associated with longer overall survival time CRC patients (P = 0.008) No significant association was observed in the *F. nucleatum* level between disease versus normal stool samples (CRC P = 0.33, CRA P = 0.15)
McCoy et al. [58]	2013	USA	Assess the abundance of Fusobacterium in the normal rectal mucosa of subjects with and without adenomas and Confirmatory Study in CRC	Tissue Adenoma (48) CRC (10)	qPCR, FISH pyrosequencing	*F. nucleatum* level is higher in adenoma subjects compared to controls (P = 0.01) No significant correlation was observed between adenoma size and *F. nucleatum* species (P = 0.57) Positive correlations were found between *F. nucleatum* species and IL-10 (r = 0.443 P = 0.01)
Kostic AD et al. [18]	2013	USA	Assessment of *F. nucleatum* status in patients with colorectal adenomas and adenocarcinomas Investigation Fn infection on cancer progression and inflammation in mouse models	Stool (56) and tissue (31)	qPCR FISH analysis	*F. nucleatum* was significantly high in adenomas compared to the normal adenomas (P < 0.004) Fusobacterium spp was high in CRC patients (P < 1 × 10^–5^) and in the stool samples with adenomas as compared to control groups (P < 5 × 10^–3^) *F. nucleatum* expands tumor-infiltrating myeloid cells in the selective manner, which can promote intestinal tumor progression and increases tumor multiplicity
Castellarin et al. [59]	2012	Canada	Evaluation of the association inflammatory microorganisms with other gastrointestinal (GI) cancers	Tissue (99)	qPCR RNA-seq	*F. nucleatum* amount was higher in tumor versus normal control (P = 2.5 * 10^–6^) Positive correlations were observed between *F. nucleatum* species and lymph node metastasis (P = 0.0035)

## Study selection

**Fig. 1:**
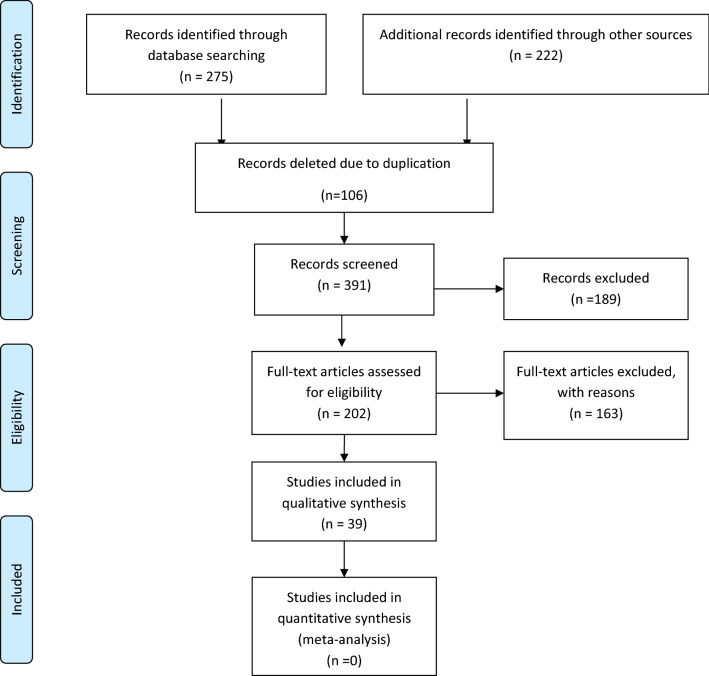
PRISMA flowchart of study selection

Three hundred and ninety one unique records were checked by title and abstract to assess their eligibility for inclusion in the project after finding a total of 497 papers and deleting the duplicate records. The full texts of 202 publications were then checked and the related articles were chosen according to the study inclusion criteria (Fig. 1). The inclusion criteria were: Studies measuring the association of *Fusobacterium nucleatum* with colorectal cancer in patients and the published studies in English language. The exclusion criteria were: 1—No access to full-text articles 2—Case reports, randomized clinical trials and review articles 3—Studies on teenagers and 4—Duplicate records were excluded. Figure 1 shows the selection process for articles. Data collected using EndNote software. The main characteristics are summarized in Table 1.

## Colorectal cancer

Cancer usually arises due to the failure of mechanisms controlling cell growth and proliferation. This control system responds to growth inhibition, growth and death signals. Colorectal carcinogenesis involves a series of well-defined changes that begin with a benign mucosal lesion called a polyp and can progress to malignancy leading to cancerous changes such as hyperplasia, adenoma, carcinoma, and metastasis [60]. The molecular mechanisms involved in these changes include activation of specific oncogenes and inactivation of tumor suppressor genes [61, 62]. Cancer is a multifactorial disease due to genetic, epigenetic, and environmental factors [63]. CRC can be asymptomatic for many years. Malignancies on the right-side of colon, including the cecum, ascending, and descending colon are associated with fatigue, weakness and iron deficiency anemia; however, left colon neoplasms are associated with concealed bleeding, alterations in bowel movement, and lower left quadrant cramp. Diagnosis is often made by the detection of fecal occult blood testing followed by endoscopy; this is then followed by biopsy and MRI [64]. One of the changes involved in the development of some cancers, including CRC, is the role of infections on tumor suppressor genes in the initiation, progression, and metastasis of cancer [65].

### *The microbiota* and colorectal cancer

Gut dysbiosis can promote CRC through various processes that include: the induction of a chronic inflammatory disease or immune response, biosynthesis of toxic metabolites and genotoxin and effect of host metabolism [66, 67]. Alternatively, Microbiota can prevent cancer by producing metabolites and enzymes. Although, some bacterial metabolites secreted from *Enterococcus faecalis*, enterotoxin *Bacteroides fragilis* or *FadA* in *F. nucleatum* are capable of damaging DNA, they can induce proliferation of colon cells in studies on gut microbiota in cancer patients [68]. Gut bacterial composition can be affected by environmental factors and tumour genomics [69]. Most cases of CRC are treatable if a diagnosis is made early enough. The survival rate in patients in whom an early detection is made is approximately 5 times greater than for patients diagnosed with advanced malignancies [70]. Consequently, it is necessary to evaluate valuable early diagnosis markers for CRC cases [70]. In the following, we will discuss the role of *F. nucleatum* as a parameter in the development and diagnosis of colorectal cancer.

### Tumorigenic potential of Fusobacterium nucleatum

Sequences of *Fusobacterium* species were found to be enriched in colorectal carcinomas [71]. The results were confirmed with the use of quantitative PCR and sequence analysis of 16S rDNA performed on 95 normal-tumor pairs of DNA. In addition, *Fusobacteria* were observed in colorectal tumors by FISH. According to the obtained results, there are some changes in the microbiota in CRC [71]. *F. nucleatum* and some common bacteria were found in the primary tumors but also in distant metastases [72]. Preliminary evidence indicates that this bacterium is initially found in cancer cells of metastasis type instead of the stroma. The tumor growth in mice with xenografts from of CRC containing *F. nucleatum* was reduced following treatment with antibiotics, consistent with the causal role played by bacteria in the development of tumors [20]. Preclinical rodent studies have recently shown that antibiotic therapy or the absence of the gut microbiota reduces the incidence of tumors in several murine colitis-associated CRC models [18]. The frequency of *Fusobacterium *in human tumors by the RNA-seq method was similar to the one obtained from mice tumors using flow cytometry [18]. In most cases, *Fusobacteria* are not part of the natural bacteria of the large intestinal flora. Studies show that cancerous tissues contain significantly more *Fusobacteria* [73]. Previous research has indicated that infection with this bacterium increases the incidence of ulcerative colitis in which inflammation of the intestinal lining destroys the intestinal cells and consequently is a risk factor for colorectal cancer [27].

### The impact of diet on the microbiota and colorectal cancer

**Fig. 2:**
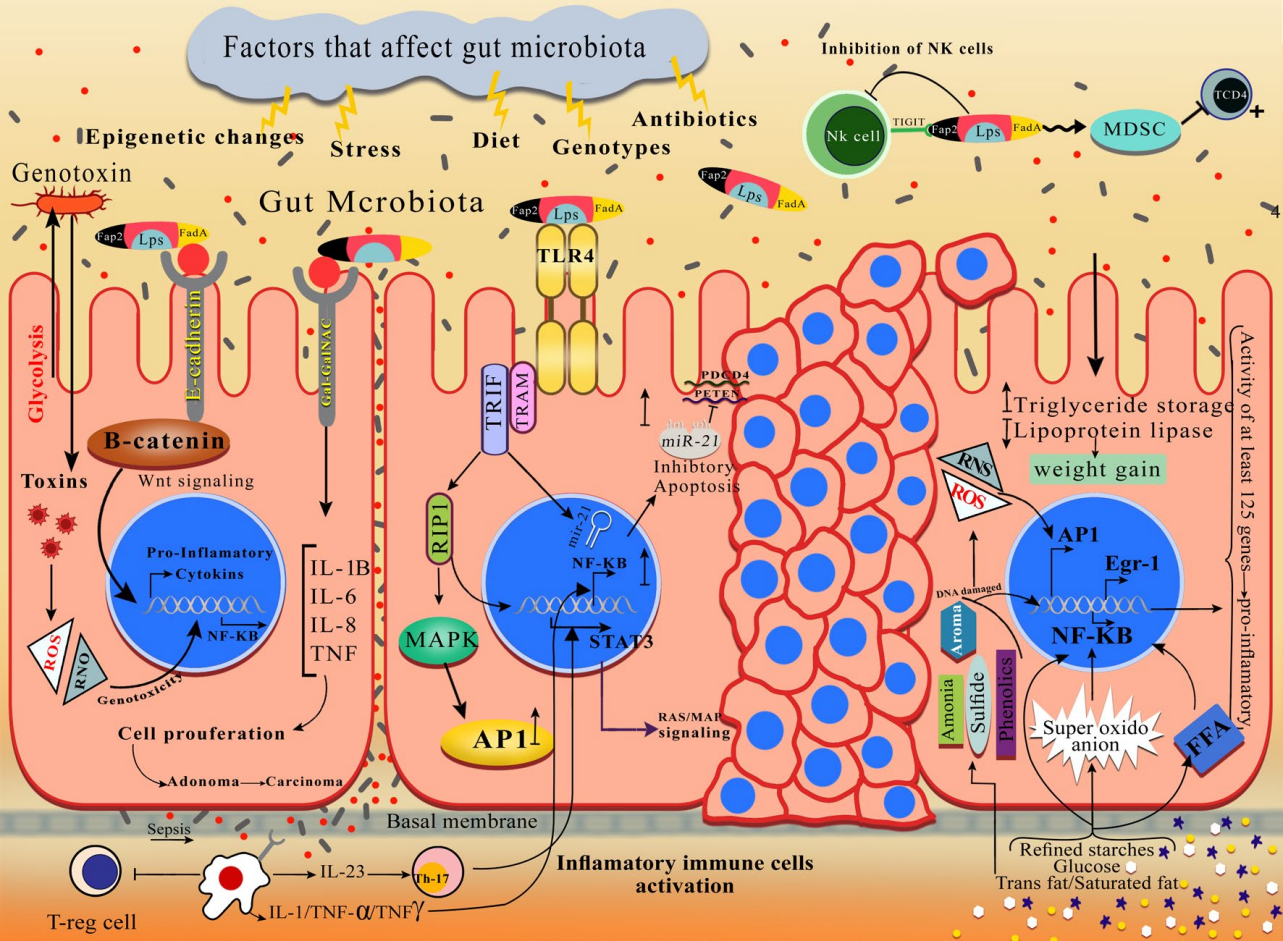
The main mechanism of *F. nucleatum* pathogenesis in CRC is illustrated. The adhesion and invasion of  *FadA *from *F. nucleatum* to epithelialand endothelial cells of human in pathway 1 can be observed while levels of  inflammatory cytokine (*IL-6, IL-8,IL-10, IL-18, TNF*-*α*, and *NF- κB*) grow in a proinflammatory microenvironment which in turn leads to colorectal tumor progression; *FadA* interaction with *E-cadherin* in pathway 2 in epithelial cells leads to  activating of  *β-catenin* signaling, increasing *NF-κB *inflammatory gene expression and enhancing tumor cell proliferation. *F.nucleatum*-infected cells, on the other hand, enhance miRNA expression by *Toll-like receptor* activation and therefore miRNA release development. *F.nucleatum* in pathways 3 and 4 reduces the activity of human T cells in a micro-suppressor of the tumor immune system. The interaction between *Fap2* from *F.nucleatum* and the human inhibitor receptor *TIGIT* in pathway 5 leads to the death of lymphocyte cells of human, resulting in a microenvironment of immunosuppression that increases the progression of CRC

At birth, four main bacterial species are present in the gut: *Firmicutes, Bacteriodetes, Proteobacteria *and* Actinobacteria* [74]. They vary greatly among healthy individuals depending on environmental, genetic, host immune system, diet, and exposure to infection or antibiotics [20, 74]. Despite the considerable variation among individuals, it has been found that there are similar microbial populations in colorectum, including anaerobic bacteria such as *Bacteroides, Eubacterium, Bifidobacterium, Fusobacterium, Peptostreptococcus, Atopobium* and optional anaerobes, including *Lactobacilli, Enterococci* and *Enterobacteria*. However, diet, age, gender, and ethnicity affect individual microbes, making its dynamic nature difficult investigate [8, 75]. From the 1990s onward, studies have shown an association between CRC and certain bacterial species [76]. Shen et al., evaluated 21 adenomas and 23 non-adenomas. In cancerous tissues, *Proteobacteria* is increased, and *Bacteroidetes* decreased [77]. It is possible that some probiotics facilitate immunomodulatory and anticancer activities in different contexts [10]. For example, lactobacillus in the lactic acid bacteria group is the main probiotic organisms. Various reports have indicated that isolates of *Lactobacillus *spp. [10] like *Lactobacillus acidophilus* in different forms may increase the anticancer effects by different mechanisms such as downregulation of *ErbB-2*, activation of natural killer cells, dendritic cell maturation, and release of probiotic-derived ferrichrome (iron-scavenging peptide) [78, 79]. The microbiome has been called “The forgotten organ” [80, 81]. Microbiota can play a key role in the development of CRC by altering the bacterial composition of the intestine (dysbiosis), high production of some bacterial enzymes, changes in the distribution of bacterial communities and alteration in bacterial metabolic activity [82, 83]. On the other hand, some of the components of the microbiota control the differentiation of intestinal epithelial cells and their proliferation, growth and development of the epithelial barrier, make strong apical bonds, protect against strains of pathogens, fermentation of carbohydrates indigestible for the production of short chain fatty acids (SCFA), bile acid metabolism and destruction of carcinogens in the diet in protection against cancer [84–87]. Many factors can alter the microenvironments of the digestive tract and consequently the bacterial flora, such as consumption of antibiotics, mental and physical stressors, radiation, and diet [88]. The microbiota play a significant role in the regulation of inflammation, immune response or hematopoiesis among others [89]. Modification of the microbiota may lead to some pathologies such as depression and cancer [90, 91]. Prevention of carcinogenesis by modulating tumor or host cell microenvironment may be possible. Moreover, the microbiota has been found to influence chemotherapy, radiotherapy and immunotherapy efficacy and toxicity [80]. *L. casei* probiotic-derived ferrichrome has its anti-tumor effect through the pathway contributing to JNK-mediated apoptosis [92]. They are also associated with decreased polarization of pro-inflammatory TH17 cells and consequently a reduction in anti-inflammatory Treg cells differentiation (regulatory T cells) and/or gut Tr1 cells (T regulatory type 1 cells) in addition to anti-inflammatory metabolites production [10, 93]. There is a special association between the microbiome profiles and cancer growth and progression. Consequently, interventions altering microbiome composition are likely to affect oncogenesis (Fig. 1). The microbiome may remain unchanged for many years. However, factors such as response to antibiotic therapy, exposure to pathogens, fasting, changes in daily diet composition and other causes such as stress, cold and diurnal rhythm disruption can cause permanent changes in it [10]. Moreover, according to reports, microbiomes affect various traits ranging from metabolism to mood [10]. The microenvironment of CRC is a complex community of genomically changed tumor cells, non-neoplastic cells, and a varied group of microorganisms [71]. Many genetic and epigenetic factors affect the reported recurrence of the disease; in many studies, the gut microbiome has not been identified as an important factor in the disease occurrence. With the progress of advanced bowel sampling techniques and analysis of both nucleic acid (RNA sequences) and protein (Proteome) products, it has been identified that the gut microbial community is a key component in not only in tumorigenesis but also the non-recurrence of disease after surgery [74, 94]. Most studies on the role of the microbiome on CRC recurrence have been investigated in clinical studies where local recurrence has occurred [74]. *F. nucleatum* can cause cancer by activating cellular signals through various mechanisms. These mechanisms are important for causing cancer in terms of cell surface receptors and their effects on the immune system (Fig. 2).

Doll and Peto have previously argued that 30% of the risk of cancer might be attributed to diet. Since then, much available evidence has pointed out that several nutrients can change cancer growth and progression [95]. Long-term dietary habits can shape the gut microbiota [87]. The International Agency for Research on Cancer (IARC), as part of the World Health Organization (WHO), has suggested that there is enough evidence to consider consumption of processed meat (Group 1) and red meat (Group 2A) in humans as having possible carcinogenic effects. IARC analyzed a group of half a million English men and women. In their analysis, they concluded that processed meat and red alcohol were related to increased risk of colorectal cancer. They also demonstrated an association between reduced risk of cancer and fiber in bread and breakfast cereals [96]. The incidence and mortality of polygenic diseases like cancer vary depending on genetic susceptibility and environmental factors. Interaction of specific nutrients on genetic code exists in all nucleated cells [93]. For example, high consumption of refined starches and sugar increases the production of superoxide anion in leukocytes, mononuclear cells and free fatty acids (FFA) [97]. It also increases the levels and activity of the nuclear factor kappa-light-chain-enhancer of activated B cells (*NF-kB*), a transcriptional regulator activating at least 125 genes, most of which are pro-inflammatory. Glucose intake also increases the two pro-inflammatory transcription factors; activated protein 1 (AP-1) and early growth response protein 1 (Egr-1) [98]. AP-1as a transcription factor induces regulation of the transcription of inflammatory cytokines, matrix metalloproteinase, as well as the transformation of apoptosis and cell proliferation [99]. Egr-1 induced endothelial gene expression [100], and modulation of transcription of tissue factor and plasminogen activator inhibitor-1 (PAI-1) [98, 101]. Increased absorption of linoleic acid, saturated fat, trans fats, refined starches and sugars can increase the production of free radicals and NF-kB activation leading to rapid expression of pro-inflammatory genes [98]. Nutrients, antioxidants, micronutrients, minerals, vitamins, coenzyme Q10 and ω3 fatty acids may inhibit NF-kB superoxide production, AP-1, and Egr-1 [98]. The evidence indicates that dietary fiber, especially starch that is resistant to digestion, enhances intestinal health. One of the issues causing the starch to be the focus of empirical research is its potential protection against CRC development [102]. Other studies have indicated that butyrate (main short-chain fatty acids) from resistant starch fermentation through the bacteria in the gut causes physiological changes in humans [103] and plays a significant role over the lifestyle in protecting the body against deteriorating metabolic control and inflammatory status associated with western lifestyles [104]. Although there is evidence of the cellular effects of butyrate, much research has been conducted to determine which mechanisms of butyrate can be used for antitumor applications [105]. Statistical and bioinformatics analysis was then performed to determine which potentially important genes and proteins are involved in inducing apoptosis of colon cancer cells. Furthermore, 1347 proteins such as isoforms of protein and modifications were detected using proteomics (2D-DIGE and mass spectrometry). Moreover, 139 proteins were identified. These proteins were likely to play a role in the apoptotic response to butyrate [93]. These reactions, along with the microbial population in the gastrointestinal tract, particularly the large intestine, cause the formation of microbiomes, including all microorganisms, their genes and metabolites. Extensive investigations to find out the genetic map of microorganisms are in progress, since microbial genes and their interactions with body cells exist before, during, and after illnesses.

## Factors affecting the intestinal microbial population

Most studies indicate that the composition of the intestinal microbiota is formed before the age of three and then has a constant composition throughout life [106, 107]. Some factors such as the use of antibiotics, special diet, and chemotherapy can interfere with the structure of the gut microbiota [108]. Since the microbiota plays an important role in the normal functioning of the body, today it is considered an organ created at birth and evolves with us. The role of the microbiota in the development of some gastrointestinal diseases has been demonstrated [109]. These diseases can range from causing inflammation to colon cancer [110]. The gut microbiota may also sometimes be involved in the development of extra-intestinal immunological diseases [110, 111]. Probiotics such as *bifidobacteria, lactobacillus, bacteroids* are mainly found in the colorectal and are beneficial for human health. They control the population of pathogenic bacteria by producing short-chain fatty acids such as acetic, butyric and propionic acid. Prebiotics are also a substrate choice for the growth of beneficial bacteria like *bifidobacteria*. Prebiotic foods include sugars such as inulin and oligofructose (FOS). During breastfeeding, the major bacterium in neonatal feces is *bifidobacteria,* but during weaning, the level of *bifidobacteria* is decreased and other bacteria such as coliform, *Clostridium* and *Streptococcus* are increased [112]. As more molecular techniques and bioinformatics analyses were developed, a better understanding of a healthy microbiome or disruption of the microbial community, including loss of beneficial bacteria or loss of diversity among them, was achieved [74]. The disorder produces a specific condition called dysbiosis, which means the loss of the health-promoting microbiome known as disease-producing pathogens. Numerous studies have shown that *Fusobacterium, Alistipes, Porphyromonadaceae, Coriobacteridae, Staphylococca-ceae, Akkermansia, Lactobacillus, Faecalibacterium, Roseburia* and *Treponema* are present in patients with CRC [58, 113–118]. The present findings emphasize the importance of cell-bacterial interactions in a network. Various mechanisms such as aberrant activation of immune cells, induction of DNA damage through production of oxygen and nitrogen species, and increased levels of immunocyte-derived bioactive molecules facilitate tumor progression [16]. Using an antibody neutralization assay, an important role for epithelial expression of *TLR2* was identified in this process [119]. These findings are consistent with the recent role of *TLR*in the development of colorectal cancer.

### Fusobacterium as a biomarker in CRC

*Fusobacterium* is a genus of anaerobic, Gram-negative, non-spore forming bacteria, similar to Bacteroides. *F. nucleatum* and *F. necrophorum* are usually members of the *Fusobacterium* species. They usually reside in the oral cavity and sometimes cause periodontal and gum infections [9]. *Fusobacterium *is considered a risk factor involved in CRC start and improvement. Immune modulation is considered to be the most important mechanism of *Fusobacterium* playing a role in CRC carcinogenesis (Fig. 1). It includes increased cells of myeloid-derived suppressor and natural killer cell inhibitors, *FadA* and *Fap2* virulence factors, microRNAs and bacterial metabolism [120, 121].

### Carcinogenesis mechanisms of F. nucleatum

Tumorigenesis mechanism of *Fusobacteria* includes receptors of pattern recognition and downstream inflammation, but these bacteria with the recruitment of myeloid cells lead to infiltration of adenomas and carcinomas, thereby resulting in *NF-κB-*dependent *TLR4* signaling [122]. Recently, it has also been demonstrated that *F. nucleatum* leads to increased expression of inflammatory mediators (IL1B, IL6 and IL8) [40, 119]. This is possibly due to miRNA-mediated activation of *TLR2/TLR4* [75, 119]. In the immune response to bacterial infection, *TLRs* are highly important. Among them, *TLR4* is considered a representative receptor for LPS. When *TLR4* is activated by LPS, a series of intracellular events are triggered. This leads to nuclear translocation of *NF-κB*, thereby increasing the expression of IL-8 gene [123]. However, *F. nucleatum *doesnot encode any known toxins, while only few common virulence factors are encoded by it. Adhesion protein *FadA* is a known virulence factor in *F. nucleatum* contributing to easier attachment and invasion of bacteria [124, 125]. *FadA* binds with an *E-cadherin* receptor and increases carcinogenesis. It causes activation of *β-catenin *and stimulates expression of transcriptional factors, *Wnt* genes, inflammatory genes, and related oncogenes (Fig. 1) [126]. In this adhesion process, *MORN2* may also be involved. However, the exact function of *MORN2* is unknown [126]. When *FadA* adhesin from *F. nucleatum* binds with *CDH1*, it causes an increase in the proliferation of *Fusobacterium*/*WNT* [126]. *FadA* and *MORN2* proteins of *F. nucleatum *play a key role in cell invasion [127]. FadA is a small ligand (125 AA) present on the surface of *Fusobacterium,* which has been shown to bind to E-Cadherin and activates *β-catenin* signaling in human cancer xenografts of mice models [112]. Thus, *FadA* binding is directly involved in host cell binding and invasion of *Fusobacterium*. *MORN2* proteins further contain a signal sequence allowing the transfer of small peptides into the periplasmic space and from the outer membrane to the extracellular environment [112]. Among the empirically identified proteins associated with disease severity is *Fap2 lectin*, a galactose adhesion protein, which binds with the NK Cell Receptor TIGIT and inhibits the destruction of tumor cells by inhibiting NK cells. Fusobacterium binds with the *Gal-GalNAc* receptor on the surface of colon cancer cells, thereby producing proinflammatory cytokines and proliferating cancer cells [128]. Metagenomic analyses have indicated increased *Fusobacterium* species in CRC compared to adjacent normal tissue by total genome sequencing, transcriptome sequencing or by 16S rRNA gene sequencing used as a tool to identify bacteria [120, 121, 129]. There has been a correlation between *F. nucleatum* in CRC, chemo-resistance and poor prognosis. According to what mentioned before, binding of the *Fap2* protein of *F. nucleatum* with the inhibitory receptor TIGIT of human protects tumors from immune cell attack expressed in natural killer (NK) cells. *F. nucleatum *would also inhibit T and NK cell activities [18]. The following section describes the other five extensive families of pattern recognition receptors (*PRRs*). They include *CLRs* (C-type lectin receptors), *LRR* (nucleotide-binding domain leucine-rich repeat) containing (*NOD-like*) receptors (*NLRs*), *RLRs* (RIG-I-like receptors), *ALRs* (*AIM2*-like receptors) and cytoplasmic DNA sensors [122]. Recent research activities emphasize the importance of pathogen–host signaling, by *PRRs* in the whole range of inflammatory responses, including cancer development and inhibition [130]. *PRRs* signaling impacts all stages of intestinal cancer, from the early stages of cancer to the metastatic stage and appearance of different cells in the tumor microenvironment, and from neoplastic cells to tumor and stromal cells [122, 130]. Small secretory peptides bind to myeloid-derived suppressor cells (*MDSCs*), thereby inhibiting CD4+ T cell. Inhibition of immune cells would be desirable for tumor cells, since it will spread the tumor to other parts of the body [112]. Investigation of 16 s rRNA sequencing of increased *F. nucleatum* levels in mucosal or fecal samples of CRC patients has shown that *F. nucleatum* levels in CRC tissue is associated with the tumor site of right-sided proximal colorectum and CpG island methylator phenotype (*CIMP*) status, microsatellite instability (*MSI*) and mutations in *BRAF, KRAS, CHD7, CHD8* and *TP53* genes [131]. Increased inflammatory cytokines such as NF-KB, TNF-α, IL10, IL8, IL6, and increased levels of *E. cadherin* on epithelial cells activates B-catenin signaling, increases *NF-κB*, C-myc expression and proliferates tumor cells [70]. Cells infected with *F. nucleatum*, due to activation of Toll-like receptors (TLR), cause more mRNA expression and release. *F. nucleatum* induces lymphocyte cell death and tumor progression by blocking G1 phase cell myeloid derivative suppressor cells (*MDSCs*) and *TIGIT* receptor inhibition [52, 132]. *F. nucleatum* also affects the *IL-6-STAT3* axis signaling and induces tumorigenesis by directly interacting with epithelial cells through activating TLRs. The key molecules stimulating tumor growth and invasion induced by these bacteria include *IL-6, cyclin D1, TNFα, MMP9* and *heparanase *[127].

### F. nucleatum, immunomodulatory of the tumor microenvironment

Cancer in its simplest form of uncontrolled cell growth in association with *F. nucleatum *is likely to affect the proliferation of cancer cells in the colorectal. According to epidemiological associations, *F. nucleatum* can improve instability and mutation of genes [56]. In the stool of mice with colon cancer, there was a correlation between immunotherapy by antibodies for *IL.10* receptor (antiIL10R) and CpG oligodeoxynucleotides with the increased *Alistipes shahii*. In this model, *A. shahii* caused an increase in the production of the tumor necrosis factor (TNF) by intrauterine myeloid cells, while TNF neutralization abolished the therapeutic effect [10]. It has been reported that enterotoxigenic *B. fragilis* stimulate pro-inflammatory Th17 cells that accelerate carcinogenesis in mice prone to the tumor [10, 79]. Compared to different bacterial strains, *F. nucleatum* can correctly identify patients with CRC. Recent studies have shown that *F. nucleatum* DNA in the early stages of the disease has the diagnostic potential as a non-invasive primary biomarker for CRC from fecal samples [52]. *Fusobacterium* is associated with the signature of human CRC gene expression. A correlation of immune cell marker genes, including tumor-associated macrophages (TAMs) (*CD209, CD206/MRC1, IL6, IL8, *and* CXCL10*), MDSCs (*CD33 *and* IL6*), dendritic cells (DCs) (*CD11c/ITGAX, CD209, TNF, *and* CD80*) and *Fusobacterium *has been found in humans [18]. Some T cell subsets are associated with CRC prognosis. For example, Th1 subsets detected by interferon-gamma secretion (IFNγ) with better prognosis and Th17 identified due to IL-17 production are accompanied by a worse prognosis. Several studies have shown that *Fusobacteria*, in particular *Fusobacterium*, is also prevalent in CRC tissues despite being predominant in the oral microbiome. Using tissues from CRC patients that were positive for the 16S ribosomal RNA gene Fuso sequence and *Th1* and *Th17* cell populations in CRC patients by flow cytometer, there was a positive relation with both *IL-17*+ and *IFNγ*+ cytokines. These findings suggest that immune responses in CRC patients (Th1 and Th17) correlate with the frequency of *Fusobacterium*, especially the *nucleatum *[56]. *Fusobacterium*-related genes, including *PTGS2* (*COX-2*), *IL1b, IL6, IL8* and *TNF*, are expressed not only in colon cancer but also in cultures of human and mouse cell lines in vitro known as the central link between inflammation and cancer [18]. In general, the expression of human *Fusobacterium*-dependent proinflammatory genes is higher in colorectal tumors than in small bowel tumors. This may be due to the anatomical location related to the fact that the listed genes are derived from human CRC [18]. Mouse studies have indicated that the gut microbiome may regulate local immune responses and affect chemotherapy and immunotherapy [74]. In patients with colorectal cancer, autophagy pathways are rich and active and high levels of *F. nucleatum *cause resistance to chemotherapy [45]. *F. nucleatum* binds with the host epithelial *E-cadherin* and stimulates colorectal carcinogenesis through *Fusobacterial* adhesion *FadA* [74]. It has also been found that *F. nucleatum* targets micro-RNA and autophagy Signaling via upregulation of *CARD3* expression causing resistance to chemotherapy [26, 45]. The direct association of *Fusobacterium* with recurrent CRC has even been postulated as a way to predict disease outcomes or change chemotherapy regimens such as inclusion of capecitabine and oxaliplatin for patients with a high burden of *F. nucleatum *[45]. These observations suggest further consideration of antimicrobial interventions as a potential treatment for patients with CRC related to *Fusobacterium *[131]. One concern is the negative effect of broad-spectrum antibiotics on the intestinal microbiome [20]. Metronidazole is ideal, since it targets various anaerobic bacteria, including *Fusobacterium* anaerobic bacteria. *Fusobacteria* are highly sensitive to metronidazole. Finally, oral administration of metronidazole to mice that were Fusobacterium- positive resulted in a significant decrease in tumor growth pathways. Treatment with metronidazole resulted in a significant reduction in the *Fusobacterium *burden [20]. However, antibiotics are somewhat similar to cytotoxic chemotherapy, and their treatment is relatively non-targeted. Enterotoxigenic *Bacteroides fragilis* (ETBF) is a toxin-producing bacterium that can activate TH_17_-mediated colitis, with simultaneous colon-specific STAT3 activation and tumor stimulation in susceptible *Apc*^*Min*^ (Multiple Intestinal Neoplasia) mice, which is reversed by IL-17 antibody blockade [133]. This issue is also considered a limitation for the treatment method. Other bacteria involved in CRC may also respond to tumor progression even beyond antibiotics. Nevertheless, as shown with metronidazole treatment, even in the late stages of the disease, it may response to clear *Fusobacterial* colonization of carcinomas in experimental mice models [134]. A recent study has shown that colorectal tumors with a high *Fusobacterium* burden are likely to recur, implying that *Fusobacterium*-positive tumors may benefit from anti-Fusobacterial treatment [20].

### Fusobacterium-associated microRNAs

MicroRNAs (MiRNAs) are non-coding molecules of RNA with approximately a length of 19–25 nucleotides. At the post-transcriptional level, they regulate target genes expression negatively. It has been shown that oncogenic miRNAs (clusters of *miR-17-92a* and *miR-25-106b* [13]) play an active role in CRC progression [135]. Moreover, it has been shown that different miRNAs such as*miR-21, miR-224, miR-200c miR-96, miR-135, miR-31, and miR-155* are related to pathogenesis of CRC [136, 137]. The microarray analysis results showed the active role of 49 *miRNAs* in *F. nucleatum* induced CRC, while in a Multi-Class-Dif analysis, there was a significant expression of 96 miRNAs in early and advanced stages of CRC with positive infection of *F. nucleatum *[13]. Among different expressions of miRNAs, *miR-4474* and *miR-4717* expressions were upregulated in CRC with positive infection of *F. nucleatum *[13]. Other genes, including *CREBBP* (*CREB*-binding protein), *STAT1, CAMK2B, PRKACB, JUN, TP53 and EWSR1,* which were involved in cancer signaling pathways were dysregulated [13]. *MiR-4802* and miR-18a* are abnormally reduced in expression by *F*. *nucleatum *that has been also known to induce chemoresistance to *oxaliplatin* and *5-FU *by reduction of apoptosis through the activation of autophagy and *TLR4/MYD88* signaling [138]. *Enrichment* of *Fusobacterium* species is observed in the microbiota in carcinomas near healthy colonic tissue. They are observed in stool samples obtained from CRC patients at a higher degree compared to healthy controls. In the early stages of tumorigenesis, *F. nucleatum *that is usually present in the oropharynx [139], is in not only carcinomas but also colorectal adenomas [21].

## Perspective and conclusion

The gut microbiota is the largest reservoir of human microbiota. They consist of species of microorganisms living in the gastrointestinal tract in coexistence with the host, reaching a population of tens of 10^14^ [140]. They include at least 1000 different species of known bacteria containing more than 3 million genes (150 times more than human genes) [140–142]. Although more than a thousand different species of bacteria are found in the human gut, only 150 to 170 of them are common in different individuals [141]. Microbiota of each person is distinctive. Therefore, the identification and determination of normal microbiota in different societies and ages are an important factor and a prerequisite for further identification of the influencing factors. A healthy and balanced gut microbiota is the key to ensuring proper digestive function [143]. They also play a crucial role in the immune system and play a vital role in a mucosal barrier [144]. Other important roles of the gut microbiota are to help digest certain foods that the stomach and small intestine cannot digest, produce some vitamins (B and K), help protect against other microorganisms and maintain intestinal integrity. In some cases, a change in the composition of the microbiota can interfere with its balance, called dysbiosis. Intestinal microbiota dysbiosis can cause intestinal diseases such as inflammatory gut disease, irritable gut syndrome, CRC and extra-intestinal diseases such as diabetes, obesity, cardiovascular disease, non-alcoholic fatty liver disease, liver cells and decreased mental health [145–148]. Researchers at the Wyss Institute at Harvard, engineered the *E. coli* strain as a probiotic-gut bacterium producing a network of nanofibers that were directly attached to the mucosa [149]. This strain fills the inflamed areas like a patch and protects these areas from environmental factors and gut microbes. This probiotic-based treatment improved rats with chemical agents and increased mucosal healing. Although many studies have focused on the local delivery of anti-inflammatory drugs to fistulae, ulcers, and intestinal inflammation, fewer studies have been conducted on mucosal healing that plays an important role in suppressing these diseases. Matrix contributes to fibrosis to repair intestinal epithelial [149]. This matrix contains Curli nanofibers, known as an intestinal barrier enhancer and an epithelial enhancer. The researchers maintain that this method can produce engineered bacteria that will settle in the gut and secrete the desired biomaterials [149]. The consumption of yogurt, kefir (a kind of yogurt drink), cheese, fruits and vegetables, seafood, avoiding foods made with preservatives and taking probiotic supplements can be effective in maintaining the normal gut microbiota balance [150]. It is still unknown whether *F. nucleatum* colonization is the outcome or a cause of carcinogenesis or inflammation in colorectal tissue. The results produced some intriguing results representing *Fusobacterium *sp. as a potential biomarker for colorectal carcinogenesis. Above all, these results provide a mechanistic insight indicative of the mediation of *Fusobacterium* sp. actions through *FadA* binding to host epithelial cells’ receptors in order to change the function of barrier, to increase inflammation through modulation of the microenvironment of tumor, and to activate pro-oncogenic signals for CRC promotion. These findings can affect the prevention, diagnosis and treatment of CRC. However, further studies are required for evaluation of the *FadA* diagnostic potentiality. More certain answers on the temporal order between *F. nucleatum *and CRC can be found in prospective studies. Even though *F. nucleatum* colonization may result from colorectal cancer, nevertheless it may play a key role in tumor malignancy increasing, metastasis promoting and antitumor immunity evasion. Some interesting questions have been raised on cancer causes based on the role of *F. nucleatum* in tumorigenesis. It is possible to reduce the development of cancer through manipulation of bacterial microbiota by fecal microbial implants, probiotics and using antibiotic treatments or vaccination. Although fecal *F. nucleatum* may be a considered as a proper measurable biological marker for detection of CRC; further research is required to make it clear how it changes in different stages of colon cancer. A combination of microbiome modulation and its products with simpler immunotherapy approaches directly targeting malignant cells could be used in the future for antineoplastic therapy. The importance of this method is in the new anticancer method or enhanced therapeutic drugs against cancer. This method will have positive clinical results for patients with cancer. Oncomicrobiotics (cocktail of bacteria or bacterial products) is a new supportive treatment for cancer improving immune responses by enhanced gut function. Therefore, much more research is needed to be conducted on cross talk between host–bacteria and their virulence proteins that play a role in colorectal carcinogenesis.

## Data Availability

Not applicable.

## Abbreviations

CRC: Colorectal cancer
DALY: Disability-adjusted life-years rates
SCFA: Short chain fatty acids
Tr1 cells: T regulatory type 1 cells
WHO: World Health Organization
FFA: Free fatty acids
NF-Kb: Nuclear factor kappa-light-chain-enhancer of activated B
AP-1: Activated protein 1
Egr-1: Early growth response protein 1
CIMP: CpGIsland methylator phenotype
MSI: Microsatellite instability
MDSCs: Myeloid derivative suppressor cells
antiIL10R: Antibodies for IL.10 receptor
TNF: Tumor necrosis factor
TAMs: Tumor-associated macrophages
IFNγ: Interferon-gamma
