# Correction: The dysbiosis signature of *Fusobacterium nucleatum* in colorectal cancer‑cause or consequences? A systematic review

**DOI:** 10.1186/s12935-022-02549-3

**Published:** 2022-03-26

**Authors:** Maryam Ranjbar, Rasoul Salehi, Shaghayegh Haghjooy Javanmard, Laleh Rafiee, Habibollah Faraji, Sima Jafarpour, Gordon A. Ferns, Majid Ghayour‑Mobarhan, Mostafa Manian, Reza Nedaeinia

**Affiliations:** 1 Applied Physiology Research Center, Cardiovascular Research Institute, Isfahan University of Medical Sciences, Isfahan, Iran; 2 Department of Genetics and Molecular Biology, School of Medicine, Isfahan University of Medical Sciences, Isfahan, Iran; 3 Pediatric Inherited Diseases Research Center, Research Institute for Primordial Prevention of Non-Communicable Disease, Isfahan University of Medical Sciences, Isfahan, Iran; 4 Molecular Medicine Research Center, Hormozgan Health Institute, Hormozgan University of Medical Sciences, Bandar Abbas, Iran; 5 Division of Medical Education, Brighton and Sussex Medical School, Falmer, Brighton, BN1 9PH Sussex UK; 6 Metabolic Syndrome Research Center, Mashhad University of Medical Sciences, Mashhad, Iran; 7 Child Growth and Development Research Center, Research Institute for Primordial Prevention of Non-Communicable Disease, Isfahan University of Medical Sciences, Isfahan, Iran

## Correction to: Cancer Cell International 21:1 (2021) https://doi.org/10.1186/s12935-021-01886-z

In the original version of this article [1], the author’s name Sima Jafarpour was incorrectly written as Sima Jafarpor. The name was displayed correctly in this correction.

Also, in subtitles “Fusobacterium as a biomarker in CRC” and “Carcinogenesis mechanisms of F. nucleatum”, Fig. 1 should be changed to Fig. 2. In the subtitle “The impact of diet on the microbiota and colorectal cancer”, the line “Consequently, interventions altering microbiome composition are likely to affect oncogenesis (Fig. 1). Figure 1 should be changed to Fig. 2.”
